# Comparison of the NEI-VFQ and OSDI questionnaires in patients with Sjögren's syndrome-related dry eye

**DOI:** 10.1186/1477-7525-2-44

**Published:** 2004-09-01

**Authors:** Susan Vitale, Linda A Goodman, George F Reed, Janine A Smith

**Affiliations:** 1 Division of Epidemiology and Clinical Research, National Eye Institute, Building 31, room 6A52, 31 Center Drive, MSC 2510, Bethesda, Maryland, USA; 2 Clinical Center, National Institutes of Health, Bethesda, Maryland, USA

## Abstract

**Background:**

To examine the associations between vision-targeted health-related quality of life (VT-HRQ) and ocular surface parameters in patients with Sjögren's syndrome, a systemic autoimmune disease characterized by dry eye and dry mouth.

**Methods:**

Forty-two patients fulfilling European / American diagnostic criteria for Sjögren's syndrome underwent Schirmer testing without anesthesia, ocular surface vital dye staining; and measurement of tear film breakup time (TBUT). Subjects were administered the Ocular Surface Disease Index (OSDI) and the 25-item National Eye Institute Vision Functioning Questionnaire (NEI-VFQ). Main outcome measures included ocular surface parameters, OSDI subscales describing ocular discomfort (OSDI-symptoms), vision-related function (OSDI-function), and environmental triggers, and NEI-VFQ subscales.

**Results:**

Participants (aged 31–81 y; 95% female) all had moderate to severe dry eye. Associations of OSDI subscales with the ocular parameters were modest (Spearman r (ρ) < 0.22) and not statistically significant. Associations of NEI-VFQ subscales with the ocular parameters reached borderline significance for the near vision subscale with TBUT (ρ = 0.32, p = .05) and for the distance vision subscale with van Bijsterveld score (ρ = 0.33, p = .04). The strongest associations of the two questionnaires were for: ocular pain and mental function with OSDI-symptoms (ρ = 0.60 and 0.45, respectively); and general vision, ocular pain, mental function, role function, and driving with OSDI-function (ρ = 0.60, 0.50, 0.61, 0.64, 0.57, and 0.67, respectively).

**Conclusions:**

Associations between conventional objective measures of dry eye and VT-HRQ were modest. The generic NEI-VFQ was similar to the disease-specific OSDI in its ability to measure the impact of Sjögren's syndrome-related dry eye on VT-HRQ.

## Background

Dry eye is a common disorder of the ocular surface and tear film and is estimated to affect from 2% to over 15% of persons in surveyed populations, depending on the definition used [1-6]. Symptoms of dry eye are a major reason to seek ophthalmic care: a study by Nelson and co-workers found that 1.3% of Medicare patients had a primary diagnosis of keratoconjunctivitis sicca or dry eye [7]. Dry eye can range from mild to severe disease; although the majority of patients with dry eye experience ocular discomfort without serious vision-threatening sequelae, severe dry eye can compromise corneal integrity by causing epithelial defects, stromal infiltration, and ulceration, and can result in visually significant scarring [8]. Moderate to severe dry eye disease can adversely affect performance of visually demanding tasks due to pain and impaired vision [9]. In addition, corneal surface irregularity due to epithelial desiccation, quantified by using corneal topography, can decrease visual acuity [10].

Patient-reported measurements used to evaluate the specific impact of eye disease and vision on symptoms (discomfort), functioning (the ability to carry out activities in daily life), and perceptions (concern about one's health) are referred to as vision-targeted health-related quality of life (VT-HRQ) instruments. Valid and reliable measurements of VT-HRQ have become essential to the assessment of disease status and treatment effectiveness in ocular disease [11]. There are two general categories of VT-HRQ instruments: generic, which are designed to be used for a broad spectrum of visual disorders and ocular disease; and disease-specific, which are tailored toward particular aspects of a specific ocular disorder. In general, disease-specific instruments tend to be more sensitive than generic ones in detecting VT-HRQ impairments [12]; however, generic instruments allow comparisons across more diverse populations and diseases [13]. In addition, generic instruments may be able to capture additional aspects of systemic disease, related to the ocular disorder in question, providing a broader characterization of health-related quality of life [14,15]. There is therefore no clear-cut basis in a given study or population for choosing a generic versus a disease-specific measure: if possible, both should be utilized to determine whether one or the other is more consistent with clinical indicators, or if one appears to obtain additional, relevant information on patient status [16]. However, it may be the case that weak-to-moderate associations between clinical indicators and quality-of-life measures indicates that the VT-HRQ measure is capturing elements of disease above and beyond those that can be measured clinically (for example, visual acuity may be good but a patient may have problems with functioning related to problems with contrast sensitivity or glare disability). Again, depending on the characterization of the disease desired and the goal of the study, a researcher might choose an instrument that either is or is not strongly correlated with clinical signs.

The measurement of the impact of dry eye on a patient's daily life, particularly symptoms of discomfort, is a critical aspect of characterizing the disease [17]. Despite the fact that most studies have found weak or no correlations between symptoms and signs of dry eye [18-20], symptoms are often the motivation for seeking eye care and are therefore a critical outcome measure when assessing treatment effect [7], and hence are increasingly used as a surrogate for ocular surface disease in many epidemiologic studies. Indeed, recent studies have focused on developing more robust ways of measuring patient-reported symptoms of dry eye [21-23]. The Ocular Surface Disease Index (OSDI)^© ^[24] was developed to quantify the specific impact of dry eye on VT-HRQ.

Sjögren's Syndrome is an autoimmune systemic disease characterized by dry mouth and dry eye signs and symptoms [25,26]. Its manifestations include fatigue, arthritis, neuropathy, and pulmonary and renal disease. Histopathologic evidence of salivary gland inflammation and the presence of serum autoantibodies SSA or SSB are important diagnostic features of the disease [27]. Sjögren's Syndrome has been stated to be the second most common autoimmune disease, ranking between rheumatoid arthritis and systemic lupus erythematosus [27]. In the U.S., it is estimated that between 1 and 4 million persons (approximately 1–2 in 200) have Sjögren's Syndrome [28]. Prevalence estimates for other countries range from 0.3 to 4.8% [29]. Female gender and older age are known risk factors for Sjögren's syndrome [30]. A wide range of studies have assessed the ocular manifestations of Sjögren's syndrome [31-33]; however, assessment of symptoms and quality of life have been limited and, in most cases, generic measures of well-being, psychological distress, and fatigue without ocular dimensions have been employed [34-40]. Further, while there are many published studies of VT-HRQ in mild to moderate dry eye, there are few publications on VT-HRQ in Sjögren's syndrome, which is characterized by dry eye causing significant ocular irritation as well as systemic disease factors that could have their own additional significant impact on VT-HRQ.

Our purpose in this study was to examine VT-HRQ in patients with primary Sjögren's syndrome, using a generic and a dry-eye-disease-specific instrument. We examined the associations of ocular surface parameters with the VT-HRQ scores, hypothesizing that the disease-specific instrument would be more closely related than the generic to the clinical markers of disease. We also examined the association of the generic and disease-specific VT-HRQ scores with each other.

## Methods

The study protocol was approved by the National Eye Institute Internal Review Board. All patients completed an informed consent prior to examination. Consecutive patients with diagnosed primary Sjögren's syndrome were recruited from the NIH Clinical Center, Bethesda, MD. The diagnosis of primary Sjögren's syndrome was based on European-American criteria, which requires at least four of the following six features: signs and symptoms of dry eye and of dry mouth, histopathologic evidence of inflammation on minor salivary gland biopsy, and positive anti-Ro or anti-La antibodies.

Before the clinical examination, a trained interviewer administered two questionnaires (described further below) to measure VT-HRQ to each patient. The subsequent clinical examination included a comprehensive anterior segment evaluation, including slit lamp biomicroscopy, evaluation of lid margin thickness and hyperemia, conjunctival erythema, chemosis, tear film debris and mucus, and extent of meibomian gland plugging. Tests of tear function and ocular surface status were performed as described below.

The OSDI [24] (provided by Allergan, Inc. Irvine, CA) was used to quantify the specific impact of dry eye on VT-HRQ. This disease-specific questionnaire includes three subscales: ocular discomfort (OSDI-symptoms), which includes symptoms such as gritty or painful eyes; functioning (OSDI-function), which measures limitation in performance of common activities such as reading and working on a computer; and environmental triggers (OSDI-triggers), which measures the impact of environmental triggers, such as wind or drafts, on dry eye symptoms. The questions are asked with reference to a one-week recall period. Possible responses refer to the frequency of the disturbance: none of the time, some of the time, half of the time, most of the time, or all of the time. Responses to the OSDI were scored using the methods described by the authors [24]. Subscale scores were computed for OSDI-symptoms, OSDI-function, and OSDI-triggers, as well as an overall averaged score. OSDI subscale scores can range from 0 to 100, with higher scores indicating more problems or symptoms. However, we subtracted the OSDI overall and subscale scores from 100, so that lower scores would indicate more problems or symptoms.

The 25-item NEI Visual Function Questionnaire (NEI-VFQ) [41,42] is a non-disease-specific (i.e., "generic") instrument designed to measure the impact of ocular disorders on VT-HRQ. Depending on the item, responses to the NEI-VFQ pertain to either frequency or severity of a symptom or functioning problem. A recall period is not specified in the questionnaire. Responses to the NEI-VFQ were scored using the methods described by the authors [43]. Subscale scores for general vision, ocular pain, near vision, distance vision, social functioning, mental functioning, role functioning, dependency, driving, color vision, and peripheral vision, as well as an overall score, were computed. The NEI-VFQ scores can range from 0–100, with lower scores indicating more problems or symptoms.

Schirmer tests of tear production without and with anesthesia were performed by inserting a Schirmer tear test sterile strip (35 mm, Alcon Laboratories, Inc, Fort Worth, TX) into the inferior fornix, at the junction of the middle and lateral third of the lower eyelid margin, for 5 minutes with the eyes closed. The extent of wetting was measured by referring to the ruler provided by the manufacturer on the envelope containing the strips. Possible scores range from 0 to 35 mm, with lower scores indicating greater abnormality in tear production. This test was repeated after instillation of topical anesthetic, 0.5% proparacaine [44]. A Schirmer without anesthesia score of ≤ 5 mm in at least one eye is one required element of dry eye, as defined by the European-American Sjögren's syndrome diagnostic criteria [45].

The assessment of ocular surface damage was performed by a cornea specialist using vital dye staining with 2% unpreserved sodium fluorescein and then 5% lissamine green dye. The corneal, temporal, and nasal regions of the conjunctiva were scored individually from 0–5 (for fluorescein) and 0–5 (for lissamine green) using the Oxford grading scheme [46]. The Oxford score was derived by adding the scores for corneal fluorescein and nasal plus temporal conjunctival lissamine green staining. Total Oxford score could range from 0–15. The van Bijsterveld score [47] (VB) was assessed using lissamine green staining of the cornea (0–3) and conjunctiva (0–3). Total VB score could range from 0–9. For all staining tests, higher scores indicate worse ocular surface damage.

Tear film stability was assessed using fluorescein tear film breakup time (TBUT). Five microliters of 2% sodium fluorescein was instilled into the inferior fornix and the patient was asked to blink several times. Using the cobalt blue filter and slit lamp biomicroscopy, the duration of time required for the first area of tear film breakup after a complete blink was determined. If the TBUT was less than 10 seconds, the test was repeated for a total of 3 values and the average was calculated.

For analysis, for each individual, the maximum (worse) score for the two eyes was used for Oxford score and VB, and the minimum (worse) score for the two eyes was used for Schirmer with and without anesthesia and for TBUT. TBUT values greater than or equal to 10 seconds [48] were coded as 10 (normal) and < 10 seconds was defined as abnormal. Schirmer without anesthesia score result of ≤ 5 mm or VB ≥ 4 were used as objective evidence of dry eye, following the European / American criteria for the diagnosis of dry eye for Sjogren's syndrome [49].

Hypotheses of specific associations were formulated based on the areas and domains assessed by the two VT-HRQ instruments. Scatterplots and Spearman's correlation coefficient (ρ) [50] were used to examine associations between pairs of variables. Multiple linear regression [51] was used to assess the strength of association between pairs of variables while adjusting for confounders (e.g., age).

## Results

### Characteristics of participants

A total of 42 patients, 40 female and 2 male, were included in this study. The average age was 55 years (range, 31–81 y). Most (81%) were of European descent. Visual acuity in the better eye was 20/20 or better for 68% of the patients; the remainder had 20/25 or better in the better eye, except for one patient who was 20/32 in both eyes. Ocular examination (Table 1) showed that, on average, the participants suffered from moderate to severe dry eye: mean Oxford score was 7.2, mean VB score was 5.3. Average Schirmer without anesthesia score was 4.8 mm, with nearly all (79%) having scores less than 10 mm and the majority (59%) having scores less than 5 mm. Mean TBUT was 2.9 seconds, with nearly all (87%) having scores less than 5 seconds.

**Table 1:** Characteristics of participants (n = 42)

	Mean, sd [range]	N (%)
Age (y)	54.9 (12.7) [31–81]	
Ethnicity		
European-derived		34 (81%)
African-derived		3 (7%)
Other		5 (12%)
Gender		
Female		40 (95%)
Male		2 (5%)
Visual acuity*		
20/20 + OU		18 (44%)
20/20+, better eye		10 (24%)
20/25+, better eye		12 (29%)
<20/25, better eye		1 (2%)
Vital dye staining		
Oxford score	7.2 (3.4) [1–14]	--
5+	--	34 (81%)
Van Bijsterveld score**	5.3 (2.7) [0–9]	--
4+	--	28 (74%)
Tear production		
Tear film break-up time (s)**	2.9 (1.7) [1–8]	--
< 5 sec	--	33 (87%)
Schirmer without anesthesia (mm)	4.9 (5.4) [0–20]	--
0–5	--	25 (60%)
5-<10	--	8 (19%)
10+	--	9 (21%)
Meibomian gland disease**		
None	--	10 (26%)
1	--	8 (21%)
2+	--	20 (53%)
European-American dry eye criteria	--	37 (90%)

*One person had missing visual acuity information; **Four persons had missing information for some components of the clinical examination

### Association of OSDI^© ^with ocular surface parameters

OSDI scores (all subtracted from 100) indicated moderate problems with symptoms, functioning, and adverse environmental conditions. Mean OSDI-symptoms score was 62.5, mean OSDI-function score was 78.2, and mean OSDI-triggers score was 60.2. However, some patients had no problems with these areas: 12% reported no problems with irritation symptoms, 21% reported no problems with functioning, and 24% had no problems with environmental triggers. Associations of the OSDI subscale and overall scores with ocular surface parameters (Oxford score, VB, TBUT, and Schirmer score with and without anesthesia) are shown in 2. In general, no substantive associations were found, except for visual functioning with TBUT (r = 0.22), and none of the observed associations reached statistical significance. Median scores on OSDI were compared between normal/abnormal categories of ocular surface variables (Schirmer without anesthesia score < 5, 5-<10, versus 10+; TFB < 5 versus > = 5; VB < 4 versus 4+, Oxford score < 5 versus 5+; European-American criteria, yes versus no). Considerable overlap in the distributions between categories was observed for all subscales, with no significant differences in median values (data not shown).

**Table 2:** Association of OSDI (scores subtracted from 100) with ocular surface parameters (Spearman ρ)

		**Oxford score**	**van Bijsterveld score**	**Tear film breakup time**	**Schirmer without anesthesia score**	**Schirmer with anesthesia score**
**OSDI**	Mean (sd); % floor					

Symptoms	62.5 (25.7); 12%	0.02	0.16	-0.10	-0.04	0.02
Visual function	78.2 (21.4); 21%	0.15	0.17	0.22	0.12	0.05
Environmental triggers	60.2 (34.3); 24%	-0.01	0.13	-0.02	0.04	0.12
Overall	70.0 (20.2); 10%	0.07	0.19	0.06	0.04	0.08

### Association of NEI-VFQ with ocular surface parameters

Overall, scores on the NEI-VFQ subscales tended to be high. Average scores for near and distance vision, social and mental functioning, dependency, driving, and peripheral vision were over 80, and a substantial percentage reported no problems at all with any of the items on the subscale: 26% for near vision, 24% for distance vision, 83% for social functioning, 17% for mental functioning, 74% for dependency, 38% for driving, and 79% for peripheral vision. The subscale indicating the most impairment was the ocular pain subscale, with a mean score of 66.7. Associations of the NEI-VFQ subscale and overall scores with ocular surface parameters (Oxford score, VB, TBUT, and Schirmer score with and without anesthesia) are shown in Table 3. Overall, associations were weak to moderate, and none attained statistical significance. General vision showed moderate correlations with Oxford score, VB, and TBUT scores (r values from 0.20–0.27). Ocular pain showed a moderate correlation with TBUT (r = 0.23) and Schirmer with anesthesia score (r = 0.22). Near vision was associated with VB (r = .20) and to a greater extent with TBUT (r = 0.32). Distance vision showed moderate associations with Oxford score, TBUT, and Schirmer with anesthesia score (r values from 0.21 – 0.26) and a stronger association with VB (r = 0.33). Social functioning was moderately associated with VB (r = .24). Role functioning was associated with Schirmer scores both with and without anesthesia, more strongly so with Schirmer with anesthesia score (r = 0.31). Dependency was associated with TBUT (r = .29) and Schirmer with anesthesia score (r = .21). An anomalous finding was that peripheral vision showed moderate association with VB score (r = .29). Mental functioning and driving showed no associations with any of the ocular surface parameters. Median scores on NEI-VFQ scales were compared between normal/abnormal categories of ocular surface variables (Schirmer without anesthesia score < 5, versus 10+; TFB < 5 versus > = 5; VB < 4 versus 4+, Oxford score < 5 versus 5+; European-American criteria, yes versus no). Considerable overlap in the distributions between categories was observed for all subscales, with no significant differences in median values (data not shown), with the exception of the European-American criteria, where, counterintuitively, scores were higher (better) for near vision for those with dry eye (45.8) than for those without (83.7; p = .03). However, only 4 patients were in the "no dry eye" category, so this result may be the consequence of unstable small sample size.

**Table 3:** Association of NEI-VFQ with ocular surface parameters (Spearman ρ)

		**Oxford score**	**van Bijsterveld score**	**Tear film breakup time**	**Schirmer without anesthesia score**	**Schirmer with anesthesia score**
**NEI-VFQ**	Mean (sd); % floor					

General vision	78.6 (12.8); 14%	0.22	0.20	0.27	-0.04	0.08
Ocular pain	66.7 (22.2); 12%	0.06	0.06	0.23	-0.06	0.22
Near vision	80.4 (19.4); 26%	0.18	0.20	0.32	-0.02	0.16
Distance vision	80.2 (18.4); 24%	0.25	0.33	0.26	-0.04	0.21
Social function	96.1 (11.2); 83%	0.14	0.24	0.15	-0.07	-0.09
Mental function	83.1 (17.5); 17%	0.15	0.18	0.19	-0.10	0.17
Role function	73.2 (25.4); 29%	0.07	-0.02	0.16	0.22	0.31
Dependency	94.4 (10.5); 74%	-0.09	-0.04	0.29	0.03	0.21
Driving	84.9 (15.5); 38%	-0.02	0.04	0.19	-0.07	-0.06
Peripheral vision	91.7 (19.6); 79%	0.11	0.29	0.15	-0.15	-0.13
Overall	83.6 (12.8); 2%	0.19	0.20	0.24	-0.04	0.19

### Association of OSDI^© ^with NEI-VFQ subscales

**Figure 1:**
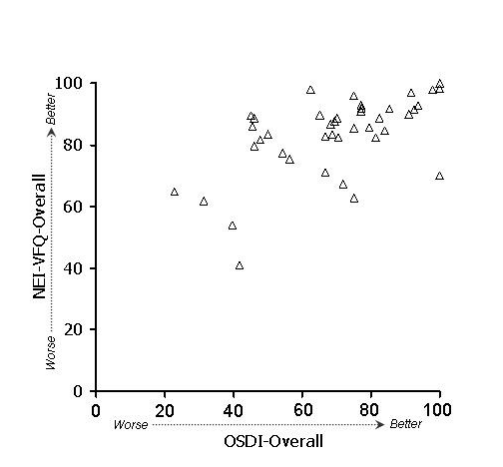
Association between OSDI (scores subtracted from 100) and NEI-VFQ overall scales. Spearman ρ: 0.61*.

**Figure 2:**
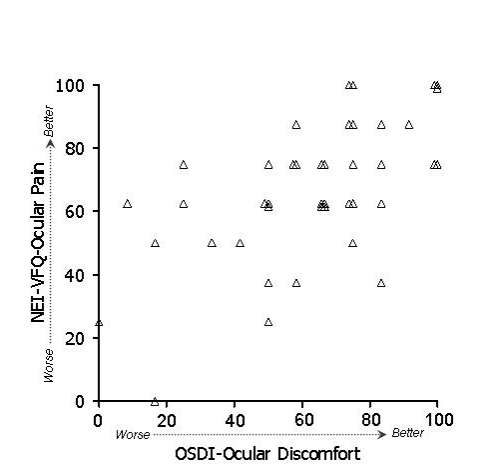
Association between OSDI ocular discomfort subscale (scores subtracted from 100) and NEI-VFQ ocular pain subscale. Spearman ρ: 0.60*

**Figure 3:**
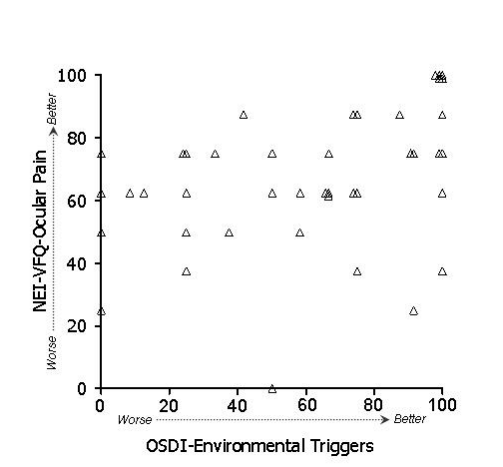
Association between OSDI environmental triggers subscale (scores subtracted from 100) and NEI-VFQ ocular pain subscale. Spearman ρ: 0.46.

**Figure 4:**
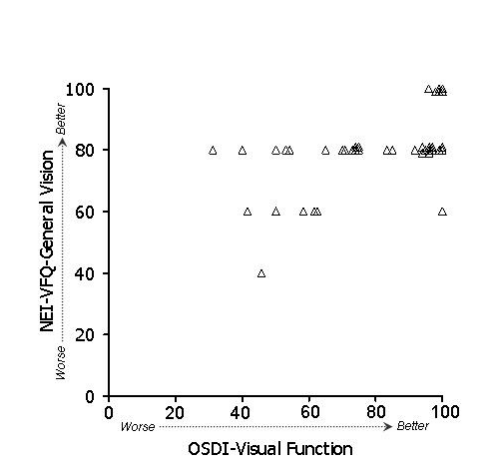
Association between OSDI visual function subscale (scores subtracted from 100) and NEI-VFQ general vision subscale. Spearman ρ: 0.61.

**Figure 5:**
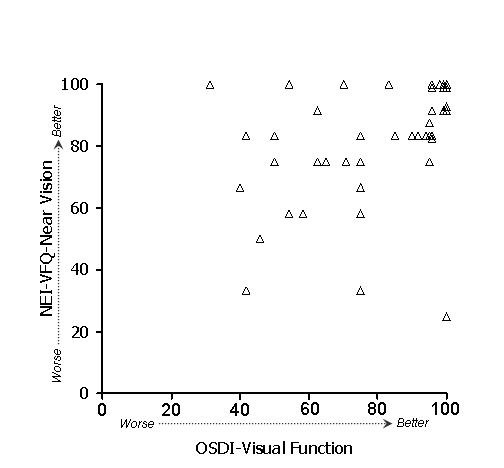
Association between OSDI visual function subscale (scores subtracted from 100) and NEI-VFQ near vision subscale. Spearman ρ: 0.46.

**Figure 6:**
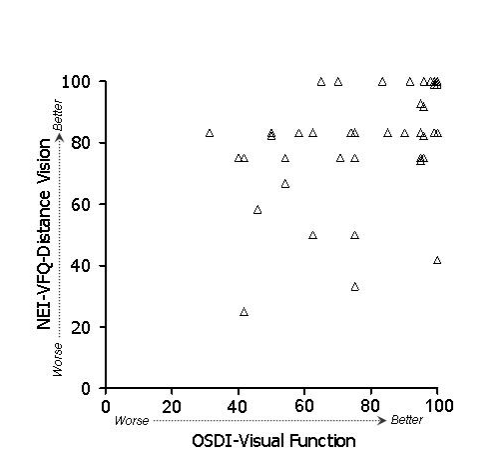
Association between OSDI visual function subscale (scores subtracted from 100) and NEI-VFQ distance vision subscale. Spearman ρ: 0.45.

**Figure 7:**
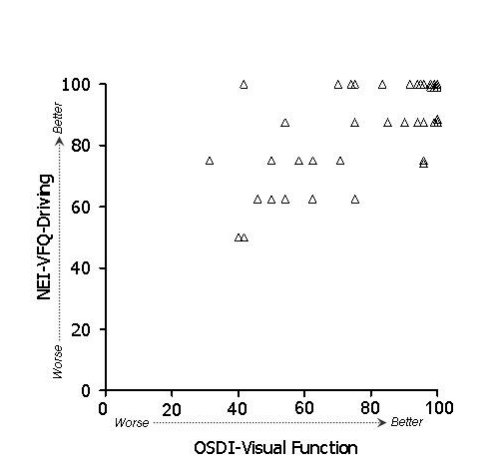
Association between OSDI visual function subscale (scores subtracted from 100) and NEI-VFQ driving subscale. Spearman ρ: 0.57.

In general, stronger associations were observed between subscales of the OSDI and NEI-VFQ (Table 4) than between ocular surface parameters and either the OSDI or the NEI-VFQ. Because of the large number of potential comparisons, we restrict discussion to associations that were hypothesized based on clinical plausibility. To test whether the overall (i.e., combined) OSDI and NEI-VFQ scales were related, we examined their linear relationship (Figure 1). Indeed, the association of these scales was strong (r = 0.61) and remained statistically significant after age adjustment. We hypothesized that the OSDI-symptoms subscale and the NEI-VFQ ocular pain subscale should show strong association, and in fact this was observed (r = 0.60, p < .001 after adjustment for age). A scatterplot of the data is shown in Figure 2. We also hypothesized that the OSDI-triggers measure should be associated with the NEI-VFQ ocular pain subscale. This association was moderate (r = 0.46, Figure 3) and did not remain statistically significant after age adjustment. The OSDI-function subscale measures a domain that has theoretical overlap with the NEI-VFQ subscales for general, near, and distance vision, as well as driving, so we hypothesized that these correlations should also be relatively strong. This was true in particular for general vision (r = 0.60, Figure 4) and driving (r = 0.57, Figure 7), both of which remained highly statistically significant after adjustment for age (p < .001). The correlations of OSDI-function with NEI-VFQ near and distance vision were not as strong (0.45, Figures 5 and 6) and were not statistically significant after adjusting for age.

**Table 4:** Associations of OSDI^© ^subscales (subtracted from 100) with NEI-VFQ subscales (Spearman ρ).

	**OSDI **Symptoms	**OSDI **Visual function	**OSDI **Environmental triggers	**OSDI **Overall
**NEI-VFQ**				

General vision	0.34	0.60*†	0.28	0.51*
Ocular pain	0.60*†	0.50*	0.46†	0.62*
Near vision	0.08	0.46†	0.23	0.33
Distance vision	0.37	0.45†	0.27	0.46
Social function	0.16	0.26	0.17	0.22
Mental function	0.45*	0.61*	0.20	0.53*
Role function	0.19	0.64*	0.33	0.48*
Dependency	0.17	0.42*	0.17	0.33
Driving	0.28	0.57*†	0.33	0.48*
Peripheral vision	0.18	0.02	0.04	0.14
Overall	0.43	0.67*	0.37	0.61*†

†Associations hypothesized at the start of the study; *statistically significant after age adjustment (p < 0.001)

Table 4 shows that, in fact, several other significant associations not conjectured in our original hypotheses were observed. In particular, the OSDI-function subscale, in addition to the associations hypothesized above, showed substantial and statistically significant associations with ocular pain (r = 0.50), mental function (r = 0.61), role function (r = 0.64), and dependency (r = 0.42). The OSDI-symptoms subscale showed a moderate and statistically significant association with NEI-VFQ mental health (r = 0.45). The overall OSDI scale showed significant associations with NEI-VFQ general vision (r = 0.51, ocular pain (r = 0.62), mental and role functioning (r = 0.53 and 0.48, respectively), and driving (r = 0.61).

## Discussion

We compared subscale scores for an ocular surface *disease-specific *instrument (OSDI) with a *generic *VT-HRQ instrument (NEI-VFQ-25) in patients with a systemic autoimmune disease associated with moderate to severe dry eye. We found that patients with primary Sjögren's syndrome had OSDI scores (mean, 30, before subtraction from 100) similar to those previously published [24] for moderate to severe dry eye patients (mean score was 36 for severe cases). Despite the fact that all of our patients had Sjögren's syndrome, with moderate to severe dry eye, we found that correlations of ocular surface parameters with VT-HRQ (i.e., patient-reported) parameters tended to be weak or nonexistent, consistent with several other studies demonstrating poor correlations between signs and symptoms of dry eye [18-20]. Indeed, contrary to our expectations, NEI-VFQ correlations with objective ocular surface parameters tended to be higher than those of OSDI, although all were relatively modest (all < 0.35) and none reached statistical significance.

One explanation could be that the nature of the items for each of these instruments is quite different. The OSDI queries the frequency of a symptom or difficulty with an activity, over a one week recall period. The NEI-VFQ incorporates questions both the frequency and intensity of symptoms and their impact on activities, with no specified recall period. Perhaps this added element of capturing both the frequency and intensity of a symptom or impact accounts for some of the differences we found. For subscales that are similar, agreement was higher but still moderate, possibly due to differences in the nature of the questions or response options. The OSDI is targeted to assess how much the symptoms of dry eye affect the patient's current status (i.e., in the past week), whereas the NEI-VFQ may be more suited to capturing the overall impact of a chronic ocular disease on VT-HRQ.

In this group of primary Sjögren's syndrome patients, associations between subscales of the NEI-VFQ and OSDI were moderate to strong (< 0.70) and in hypothesized directions. Significant associations were seen between OSDI and NEI-VFQ overall scales; OSDI-symptoms and NEI-VFQ ocular pain; and OSDI-function and NEI-VFQ general vision and driving. This suggests that both instruments are capturing important aspects of VT-HRQ. It is not surprising that the highest correlations were observed between subscales with similar domains, which serves to validate the use of alternate methodologies. On the other hand, it is counter-intuitive that the generic and disease-specific instruments appeared similar (or that the generic seemed to do a little better) with respect to their association with objectively measured clinical signs of dry eye, as the NEI-VFQ was designed to capture broader aspects of VT-HRQ. For the NEI-VFQ, we found moderate correlations (greater than 0.3) of distance vision with VB and near vision with TBUT. This was surprising, as one may have expected that subscales measuring ocular discomfort or pain (i.e., more disease-specific for dry eye) would have the strongest correlations with clinical measures of dry eye. Clinical signs of dry eye include measures of tear production, ocular surface staining, and tear film break-up; visual acuity and other aspects of visual function are not generally as widely used. However, some investigators have reported that visual acuity in dry eye patients is correlated with decreased spatial contrast sensitivity [52] and is functionally reduced with sustained eye opening due to increased surface irregularity which can be detected with corneal topography [53,10], which could explain our finding of moderate associations of ocular surface measures with near and distance vision. It has been proposed [10] that "subtle visual disturbance" is an important reason for dry eye patients to seek care. Indeed, improvement in blurred vision symptoms was one of the most frequently reported benefits of topical cyclosporine treatment for dry eye in a large, multicenter clinical trial [54]. The impact of the quality of vision or functional visual acuity on VT-HRQ has not been a focus of studies of the subjective aspects of dry eye. Our data indicate that the impact of dry eye on VT-HRQ is only partially accounted for by ocular pain in patients with severe dry eye, such as in Sjogren's syndrome.

Would we expect the associations to be different in Sjögren's patients? Sjögren's syndrome is an autoimmune exocrinopathy and effects of its systemic nature and chronicity on dry eye may have been more readily captured by the NEI-VFQ's ability to measure both frequency and intensity of problems with VT-HRQ. In contrast, although the OSDI includes items to measure function, responses are limited to the frequency of problems. Because the type of dry eye in Sjögren's syndrome is more likely to be severe, and all patients in our study had Sjögren's-related dry eye, we speculated that somewhat stronger associations between signs and symptoms might be observed. On the other hand, ocular surface inflammation and decreased corneal sensation are features of severe dry eye which might alter a patient's perception of symptoms of ocular irritation and might be the cause of weaker correlations between signs and symptoms [48,55]. Indeed, reduced corneal sensation could provide inadequate feedback through the ophthalmic nerve to the central nervous system, resulting in less efferent stimulation to the lacrimal gland with reduced tear production and promotion of a vicious cycle. In addition, meibomian gland dysfunction plays a key role in dry eye in Sjögren's syndrome [56]. Therefore, aqueous and evaporative tear deficiency may combine to produce a particularly diseased ocular surface.

## Conclusions

In addition to clinical signs, it is important to include assessments of VT-HRQ and visual function to fully characterize the impact of dry eye on health status. The correlation between signs and VT-HRQ are modest at best, indicating that VT-HRQ is capturing an additional component of disease that is not captured by the clinical assessment. This does not necessarily mean that the measures of VT-HRQ or the methods of detecting clinical signs are deficient, but rather that VT-HRQ is an additional element of the overall impact of this disease process on affected individuals. Furthermore, in diseases with systemic manifestations, such as Sjögren's syndrome, that may have an influence on quality of life independent of dry eye symptoms, appropriate tests of VT-HRQ are critical to completely characterize quality of life in these patients. It may also be valuable to explore possible differences in associations of clinical signs with VT-HRQ in patient populations with different manifestations or causes of dry eye.

## List of abbreviations

VT-HRQ: Vision-targeted health-related quality of life; TBUT: Tearfilm breakup time; OSDI: Ocular Surface Disease Index; NEI-VFQ: National Eye Institute Visual Function Questionnaire; VB: van Bijsterveld

## Authors' contributions

SV helped to design the study and performed all analyses and took the lead in writing the manuscript. LG performed the patient interviews and assisted with data analyses. GFR provided advice on statistical methods and presentation of the results. JA conceived and helped to design the study and assisted with writing the manuscript.
